# Comprehensive Metabolomic–Transcriptomic Analysis of the Regulatory Effects of *Armillaria mellea* Source Differences on Secondary Metabolism in *Gastrodia elata*

**DOI:** 10.3390/biology15020196

**Published:** 2026-01-21

**Authors:** Duo Han, Chengcui Yang, Liuyuan Bao, Li Dong, Haiyan He, Peng Tang, Yongzhi Zhang, Fen Xiong, Honggao Liu, Shunqiang Yang

**Affiliations:** 1 College of Agronomy and Life Sciences, Zhaotong University, Zhaotong 657000, China; 18887212025@163.com (D.H.); ycjgxcm@163.com (C.Y.); 47015@ztu.edu.cn (L.B.); 14769158780@163.com (L.D.); 34017@ztu.edu.cn (H.H.); tangpeng@ztu.edu.cn (P.T.); 34006@ztu.edu.cn (Y.Z.); 202215060131@stu.ztu.edu.cn (F.X.); 2 College of Agronomy and Biotechnology, Yunnan Agricultural University, Kunming 650201, China

**Keywords:** *Armillaria mellea*, *Gastrodia elata*, metabolomics, transcriptomics, gastrodia-fungus interaction

## Abstract

*Armillaria mellea* (*A. mellea*) serves as a crucial nutritional source for *Gastrodia elata* (GE) growth, and its origin directly influences the GE quality and yield. This study analyzed GE symbiotic with *A. mellea* from different sources using metabolomics and transcriptomics. Results demonstrated that Group A exhibited significant differences in metabolites and gene expression compared to other groups. Group A showed significantly higher accumulation of active components like gastrodin and p-hydroxybenzyl alcohol than others, but its yield was lower than Group B. Metabolomic analysis identified 2418 metabolites, while transcriptomic sequencing produced 964,110,904 clean reads, with 14,637 annotated transcripts. KEGG analysis revealed that Group A’s DEGs and DEMs were co-enriched in three key pathways, including flavonoid biosynthesis, phenylpropanoid biosynthesis, and plant hormone signal transduction, such as the positive regulatory roles of key genes (*CHS*, *4CL*, *MYC2*) on metabolites such as hesperetin, ferulate, and jasmonic acid, respectively. The coordinated upregulation of gene–metabolite interactions in Group A GE may be closely related to the accumulation of major active components, indirectly suggesting the influence of the *A. mellea* source on metabolic and transcriptional response differences in GE. This study, centered on the host GE, indirectly deduces the association between *A. mellea* and GE, providing a theoretical basis for screening high-quality “fungus-GE” combinations. Further in-depth research and validation experiments will be conducted in conjunction with fungal omics.

## Simple Summary

*Gastrodia elata*, as a plant with dual purposes for medicine and food, has garnered significant attention. However, the formation of its quality and yield is influenced by differences in the sources of *Armillaria mellea*. This study aims to reveal the varying effects of *Armillaria mellea* from different sources on the secondary metabolism of co-cultivated *Gastrodia elata*, which holds substantial implications for the *Gastrodia elata* cultivation industry. Centered on the host (*Gastrodia elata*), the study indirectly deduced that *Armillaria mellea* A may induce the upregulation of numerous metabolites and genes in *Gastrodia elata*, thereby promoting the accumulation of beneficial components, while *Armillaria mellea* B might be more conducive to enhancing the yield. The co-enrichment pathway analysis of flavonoids, phenylpropanoids, and plant hormone signal transduction identified multiple key regulatory genes (such as *CHS* and *4CL*) and key metabolites (such as hesperetin and ferulate) in *Gastrodia elata*. Our findings provide important insights for the next steps in screening and validating high-quality regulatory genes. Based on these conclusions, further in-depth research will be conducted in combination with a fungal omics analysis, with the aim of providing molecular evidence for constructing efficient “fungus-Gastrodia” combinations.

## 1. Introduction

*Gastrodia elata* (GE) is a rootless, leafless, non-photosynthetic, mycoheterotrophic perennial herb belonging to the Orchidaceae family, which has garnered significant attention due to its status as a medicinal and edible plant [1]. The primary constituents of GE include aromatic compounds, organic acids, steroids, sugars, and glycosides [2]. Known bioactive substances responsible for its medicinal properties include gastrodin (GAS), p-hydroxybenzyl alcohol (HBA), parishins, and polysaccharides. Among these, GAS and HBA are recognized as phytochemical markers in the Chinese Pharmacopoeia for the quality control of GE [3]. The active components of GE exhibit diverse pharmacological activities and are widely used in clinical applications [4,5].

During the seed germination and growth process of GE, it forms symbiotic relationships with germination fungi (*Mycena* spp.) and Armillaria mellea (*A. mellea*), respectively, resulting in the formation of sexually propagated corms and asexually developed tubers. These fungi serve as the nutritional source for GE [6]. The interaction between *A. mellea* and GE is mediated by strigolactone-induced reactive oxygen species changes [7], playing a pivotal role in the quality and yield of GE. These two metrics have also become key criteria for screening high-quality *A. mellea* [8]. During the asexual propagation stage, the growth rate, hyphal branching ability of *A. mellea*, and its symbiotic relationship with GE significantly influence the yield and quality of GE [9,10]. Multiple studies have shown that the contents of major active components in GE, such as GAS, HBA, and polysaccharides, vary depending on *A. mellea* [11,12]. Among these, GAS increases significantly from corms to tubers [13]. High-quality *A. mellea* can also notably enhance the trace element content in GE and promote high yields [14]. Thus, the selection of fungal strains directly affects the quality and yield of GE, and identifying and screening highly efficient symbiotic strains is a critical technological bottleneck for improving GE production and quality. Field investigations have revealed that farmers in the same region purchase *A. mellea* from diverse sources, leading to significant variations in GE yields and unknown quality levels. Therefore, understanding the varying effects of *A. mellea* from different sources on the growth and development of GE is crucial for advancing the GE industry.

Currently, metabolomics and transcriptomics are extensively employed in identifying plant components and investigating molecular mechanisms [15]. These approaches offer effective strategies for elucidating the molecular basis of active compound formation and enable the precise identification of genes involved in complex biological processes [16]. Omics technologies have also been extensively applied in research related to GE. Metabolomics has been widely employed to evaluate the therapeutic effects of GE on cardiovascular, cerebrovascular, and nervous system-related diseases [17,18], as well as to analyze metabolic profiles post-processing [19] and assess quality [20,21]. Transcriptomics has been used to reveal the expression of developmental regulators in GE [22], elucidate the metabolic characteristics of symbiotic GE and its growth [23], and identify key regulatory genes that enhance the content of active components such as GAS [24]. The combined application of metabolomics and transcriptomics has been utilized to assess GE’s pharmacological effects [25] and perform comprehensive analyses of its nutritional characteristics and biosynthetic molecular mechanisms [26]. These approaches provide mature and advantageous technical support for this study.

Current research predominantly focuses on the metabolism and transcription of GE itself, lacking a systematic comparison of the effects of *A. mellea* from different sources on GE. This study integrates metabolomics, transcriptomics, and major active components of GE to identify key metabolites, regulatory genes, and metabolic pathways in GE cultivated with *A. mellea* from various sources. Additionally, we construct a correlation network between differentially expressed genes (DEGs) and differentially metabolites (DEMs), aiming to provide a scientific basis for selecting high-quality strains and improving the quality of GE cultivation.

## 2. Materials and Methods

### 2.1. Cultivation and Harvesting of GE Materials

Five different *A. mellea* (A–E) were procured from various production companies (Supplementary Materials; Table S1) and were used in companion planting trials with the same GE seeds. The cultivation site was located in Xiaocaoba, Yiliang, Zhaotong, Yunnan, China, with coordinates at longitude 104°3′21.5665″ E, latitude 27°37′27.3958″ N, and an altitude of 1185 m. Except for the *A. mellea* strains, all other materials and management practices were kept consistent. We have outlined the detailed cultivation procedures in the Supplementary Materials (Table S2 and Figure S1).

During the mature stage, the tubers of GE associated with different sources of *A. mellea* were harvested separately to calculate the yield per unit area. Fresh GE was washed with distilled water to remove surface contaminants, air-dried on sterile absorbent paper, and then cut into approximately 0.5 cm^3^ pieces using a sterilized scalpel. The pieces were rapidly frozen in liquid nitrogen and finely ground into a homogeneous powder, stored in cryotubes, and preserved at −80 °C for metabolomic and transcriptomic analyses, with three biological replicates. Additionally, 10 individual tubers weighing between 150 and 200 g from each treatment group were selected, cleaned, steamed for 20 min until just cooked through, dried to a constant weight in a 60 °C oven, and then crushed and sieved through an 80-mesh sieve for quantitative component analysis, with three technical replicates. All molecular analyses in this study were conducted on GE tissues, with no genomic or transcriptomic sequencing performed on *A. mellea*.

### 2.2. Quantification of Main Active Components in GE by HPLC

Preparation of Test Solution: Precisely weigh 0.20 g of GE powder and place it in a 5.00 mL volumetric flask. Dilute to the volume with a 75% ethanol (Sigma-Aldrich Shanghai Trading Co., Ltd., Shanghai, China) solution, seal, and ultrasonically extract at 50 °C for 30 min. After ultrasonic extraction, replenish the lost volume with 75% ethanol, and filter through a 0.22 μm organic microporous membrane to obtain the test solution. Quantify using HPLC (LC-40DXR, Shimadzu, Japan) under the following conditions.

HPLC Conditions: column: Inertsustain C18 (4.6 mm × 250 mm, 5.0 μm); mobile phase: acetonitrile (Sigma-Aldrich Shanghai Trading Co., Ltd., Shanghai, China) (A), 0.1% formic acid (Sigma-Aldrich Shanghai Trading Co., Ltd., Shanghai, China) in water (B), gradient elution (0–15 min, 2–5% A; 15–25 min, 5–12% A; 25–45 min, 12–18% A; 45–60 min, 18–25% A; 60–62 min, 25–95% A); flow rate: 1 mL·min^−1^; injection volume: 5 μL; column temperature: 40 °C; detection wavelength: 270 nm. The concentration of characteristic components is determined using the standard solution’s regression equation.

Preparation of standard solutions and construction of standard curves: Accurately weigh appropriate amounts of GAS, HBA, PHBA, HBD, and parishin (A, B, C, E) (Solarbio Science & Technology Co., Ltd., Beijing, China) respectively, dissolve them thoroughly in 75% ethanol to prepare reference solutions with concentrations of GAS at 0, 0.05, 0.1, 0.2, 0.25, 0.5, 1.0, and 2.0 mg·mL^−1^; HBA, PHBA, HBD, at 0, 0.00125, 0.0025, 0.00625, 0.0125, 0.025, 0.05, 0.1 mg·mL^−1^; and parishin (A, B, C, E) at 0, 0.0025, 0.00625, 0.0125, 0.025, 0.05, 0.1, 0.25 mg·mL^−1^. Analyze these solutions under the same chromatographic conditions and establish regression equations (Supplementary Materials; Table S3). 

### 2.3. Determination of Crude Polysaccharides and Monosaccharide Components

#### 2.3.1. Extraction and Purification of GE Crude Polysaccharide

Disperse GE powder in pure water at a mass-to-volume ratio of 1:40, heat and stir at 60 °C for 1.5 h, allow to stand at low temperature for 4 h, then centrifuge to remove water-insoluble components. Collect the supernatant and add a large volume of ice-cold ethanol at a volume ratio of 1:5 to precipitate crude GE polysaccharides. Collect the precipitate and dry it to obtain the initial product. Next, dissolve the initial product in pure water, take 4 mL of the solution and mix it with 1 mL of chloroform-n-butanol (prepared as a 4:1 volume ratio mixture), shake vigorously for 30 min, and centrifuge to separate the aqueous and organic phases. Add the same volume of chloroform-n-butanol (Sigma-Aldrich Shanghai Trading Co., Ltd., Shanghai, China) solution to the aqueous phase and repeat the process, performing a total of six repetitions to remove proteins from the initial product. Mix the resulting aqueous phase with activated carbon, shake thoroughly, and allow it to stand until the aqueous phase becomes colorless. Filter to collect the aqueous phase and freeze-dry to obtain crude GE polysaccharides. Weigh the mass and calculate the polysaccharide extraction yield.

#### 2.3.2. Determination of Molecular Weight of GE Crude Polysaccharides

After verifying that the crude GE polysaccharides contained over 85% purity, high-performance gel permeation chromatography (HP-GPC) was utilized with an aqueous mobile phase to determine the molecular weights of the various GE polysaccharides (Supplementary Materials; Figure S2).

#### 2.3.3. Analysis of Monosaccharide Components in GE Crude Polysaccharides

For different GE crude polysaccharides, trifluoroacetic acid (Sigma-Aldrich Shanghai Trading Co., Ltd., Shanghai, China) was used to hydrolyze them into monosaccharides at 121 °C for 2 h. An ion chromatograph (ICS-600, Thermo Fisher Scientific (China) Co., Ltd., Shanghai, China) was employed to detect and quantify the presence of 13 types of monosaccharides, including fucose, rhamnose, arabinose, galactose, glucose, fructose, ribose, xylose, mannose, galacturonic acid, glucuronic acid, mannuronic acid, and guluronic acid. Regression curve parameters are shown in Table S4. The monosaccharide TIC chromatograms of sample and reference standard are shown in Figure S3.

### 2.4. Broad-Targeted Metabolomics Analysis

#### 2.4.1. Sample Extraction

The GE samples were freeze-dried in a vacuum freeze dryer (Scientz-100F, Ningbo Xinzhi Biotech Co., Ltd., Ningbo, China) for 63 h and then pulverized using a grinding machine (MM 400, Retsch, Haan, Germany) at 30 Hz for 1.5 min. Using a precision electronic balance (MS105DΜ, Mettler-Toledo (Shanghai) Co., Ltd., Shanghai, China), 50 mg of the powdered sample was weighed and combined with 1200 μL of a pre-chilled 70% methanol–water internal standard extraction solution at 20 °C. The mixture was vortexed at 30 min intervals for 30 s each cycle, repeated six times. Following centrifugation (12,000 rpm, 3 min), the supernatant was collected, filtered through a 0.22 μm microporous membrane, and stored in sample vials for UPLC-MS/MS analysis.

#### 2.4.2. Chromatographic Conditions

The data-acquisition instrument system primarily consists of ultra-performance liquid chromatography (UPLC) (ExionLC™ AD, https://sciex.com.cn/) and tandem mass spectrometry (MS/MS). Liquid chromatography conditions: column: Agilent SB-C18 (1.8 µm, 2.1 mm × 100 mm). Mobile phase: phase A: ultrapure water (containing 0.1% formic acid); phase B: acetonitrile (containing 0.1% formic acid). Elution gradient: 0.00–9.00 min: Linear increase from 5% to 95% Phase B. 9.00–10.00 min: maintained at 95% Phase B. 10.00–11.10 min: reduced to 5% Phase B. 11.10–14.00 min: equilibrated at 5% Phase B. Flow rate: 0.35 mL·min^−1^. Column temperature: 40 °C. Injection volume: 2 μL.

#### 2.4.3. Mass Spectrometry Conditions

The electrospray ionization (ESI) source temperature was set at 500 °C; the ion spray voltage (IS) was 5500 V (positive ion mode)/–4500 V (negative ion mode). The ion source gas I (GSI), gas II (GSII), and curtain gas (CUR) were set at 50, 60, and 25 psi, respectively, with the collision-induced ionization parameter set to high. QQQ scanning was performed in MRM mode, with the collision gas (nitrogen) set to medium. The declustering potential (DP) and collision energy (CE) for each MRM ion pair were optimized through further adjustments. Based on the metabolites eluted in each period, a specific set of MRM ion pairs was monitored for each period.

#### 2.4.4. Metabolite Identification and Quantification

Based on the MWDB (Metware Database), metabolites were identified using secondary spectral information, and quantitative analysis was performed using the multiple reaction monitoring (MRM) mode of the triple quadrupole mass spectrometer. After obtaining the metabolomic data from different samples, the peak areas of all chromatographic peaks were integrated, and the mass spectral peaks of the same metabolite across different samples were corrected for integration [27].

### 2.5. RNA Sequencing and Bioinformatics Analysis

#### 2.5.1. RNA Extraction and Detection

The GE samples were extracted using the ethanol precipitation method and CTAB-PBIOZOL. The successfully extracted RNA was dissolved in 50 µL of DEPC-treated water. Subsequently, the total RNA was identified and quantified using the Qubit fluorometer (Thermo Fisher Scientific, Singapore) and the Qsep400 high-throughput biological fragment analyzer (BiOptic Inc., Taiwan, China).

#### 2.5.2. mRNA Library Construction

By leveraging the structural feature that most eukaryotic mRNAs possess a poly-A tail, poly-A-tailed mRNAs are enriched using oligo (dT) magnetic beads. A fragmentation buffer is then introduced to break the RNA into short fragments. These fragments serve as templates for synthesizing the first cDNA strand with random hexamer primers. Next, buffer, dNTPs (dTTP, dATP, dGTP, and dCTP), and DNA polymerase are added to synthesize the second cDNA strand. The resulting double-stranded cDNA is purified using DNA purification magnetic beads. The purified cDNA undergoes end repair, A-tailing, and sequencing adapter ligation. Fragments are then size-selected using DNA purification magnetic beads, followed by PCR amplification to yield the final cDNA library. After library construction, the concentration is quantified using a Qubit fluorometer, and fragment size distribution is analyzed with the Qsep400 high-throughput bioanalyzer (BiOptic Inc., Taiwan, China).

#### 2.5.3. Sequencing and Analysis

Once the libraries pass quality control, they are pooled based on the target data volume and sequenced using the Illumina platform. The sequencing process follows the fundamental principle of Sequencing by Synthesis. The instrument captures fluorescent signals in each sequencing cluster in real-time and converts them into base sequences.

The raw sequencing data underwent processing through the following workflow: (1) quality control: the raw data were filtered using fastp to remove adapter-containing reads, resulting in “Clean Data” [28]. (2) Alignment: the Clean Data were aligned to the specified reference genome to generate “Mapped Data” [29]. (3) Novel transcript prediction: StringTie was used for novel gene prediction and counting. StringTie employs a network flow algorithm and optionally de novo assembly to assemble transcripts [30]. (4) Gene expression quantification: featureCounts was used to calculate gene alignment counts, followed by computing each gene’s FPKM based on gene length [31]. (5) Differential expression analysis: DESeq2 (version 1.22.1) was employed to analyze differential expression between two groups, with *p* values adjusted using the Benjamini and Hochberg method. The adjusted *p* values and log2 fold-changes were used as thresholds for significant differential expression [32,33]. (6) Differential gene enrichment analysis: KEGG enrichment analysis was conducted based on hypergeometric testing, performing hypergeometric distribution tests on pathways for differential expression analysis [34].

### 2.6. Statistical Analysis

We utilized a free online data analysis platform (https://cloud.metware.cn/) to conduct principal component analysis (PCA), generate cluster heatmaps, and perform correlation network analysis on both metabolomic and transcriptomic datasets. Identified metabolites were annotated using the KEGG Compound database (http://www.kegg.jp/kegg/compound/, accessed on 8 July 2024), and annotated metabolites were then mapped to the KEGG Pathway database (http://www.kegg.jp/kegg/pathway.html, accessed on 8 July 2024) [35]. The Benjamini–Hochberg (BH) method was employed for multiple testing correction in metabolomics, transcriptomics, and pathway enrichment analyses. Mass spectrometry data were processed using Analyst 1.6.3 software. Content bar charts and differential analysis were plotted using Origin 2024 software, with significant differences determined via Duncan’s multiple range test at a significance level of *p* < 0.05. The cDNA libraries were sequenced on the Illumina sequencing platform by MetWare Biotechnology Co., Ltd. (Wuhan, China).

## 3. Results

### 3.1. Analysis of Main Active Components and Yield in GE Cultivated with A. mellea from Different Sources

**Figure 1:**
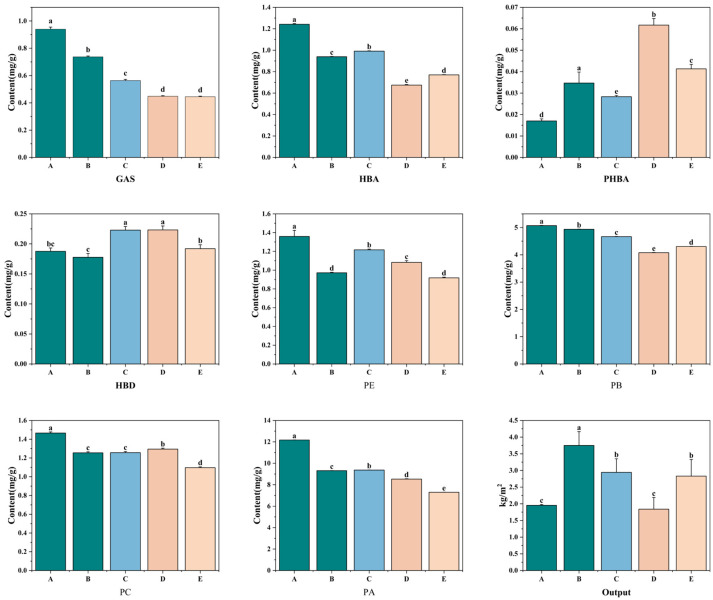
Analysis of the contents and yields of main active components in *Gastrodia elata* cultivated with *Armillaria mellea* from different sources (*n* = 3). A–E: Five groups of *Gastrodia elata* cultivated with *Armillaria mellea* from different sources; GAS: gastrodin; HBA: p-hydroxybenzyl alcohol; PHBA: p-hydroxybenzoic acid; HBD: p-hydroxybenzaldehyde; PA: parishin A; PB: parishin B; PC: parishin C; PE: parishin E; a–e denote groups that differ significantly at the 0.05 level according to Duncan’s test.

The contents of major active components—GAS, HBA, p-hydroxybenzoic acid (PHBA), p-hydroxybenzaldehyde (HBD), parishin A (PA), parishin B (PB), parishin C (PC), parishin E (PE)—and yields in GE tubers cultivated with five *A. mellea* were analyzed (Figure 1; Supplementary Materials; Table S5). Results revealed variations in the main active components and yields among the groups. Group A exhibited the highest levels of GAS (0.94 mg·g^−1^), HBA (1.36 mg·g^−1^), and parishins, with the total parishin content reaching 20.07 mg·g^−1^. The combined content of GAS and HBA far exceeded the 0.25% threshold specified in the 2025 edition of the Chinese Pharmacopoeia, indicating superior quality. Group D showed the highest PHBA content (0.0617 mg·g^−1^), while Groups C and D had the highest HBD content (0.223 mg·g^−1^). In terms of active components, Group A demonstrated the best quality. However, its yield was lower than other groups, with Group B achieving the highest average yield (3.75 kg·m^−2^).

### 3.2. Analysis of Polysaccharides and Monosaccharide Composition in GE Cultivated with A. mellea from Different Sources

To elucidate the differences in polysaccharides derived from GE symbiotically associated with various *A. mellea*, this study employed a water extraction and alcohol precipitation method to extract polysaccharides from GE tubers. Subsequently, the proteins were removed using the Sevage method six times, yielding five groups of crude polysaccharides. The purity test results all showed values ≥ 85%. The extraction rates for groups A–E were calculated as 7.33%, 4.00%, 5.00%, 10.66%, and 10.66%, respectively (Table 1).

The extracted polysaccharides were analyzed via gel permeation chromatography (GPC) (Table 2). The results showed that the number-average molecular weight (Mn) of A was 12,091 g·mol^−1^ (accounting for 35.56%) and 866 g·mol^−1^ (accounting for 30.44%), B had an Mn of 87,128 g/mol (accounting for 100.00%), C had an Mn of 284,616 g·mol^−1^ (accounting for 52.39%), D had an Mn of 72,441 g·mol^−1^ (accounting for 100.00%), and E had an Mn of 12,767 g·mol^−1^ (accounting for 50.39%) and 851 g·mol^−1^ (accounting for 44.49%). The polydispersity index (PDI) of A, C, and E tended to be closer to 1, indicating a narrower molecular weight distribution and relatively smaller overall molecular weights.

Analysis of the monosaccharide components of GE polysaccharides revealed four main monosaccharides: glucose (Glc), galacturonic acid (Gal-UA), galactose (Gal), and arabinose (Ara) (Table 1). Glc was the most abundant monosaccharide by mass, with the highest content in Group A at 668.08 µg·mg^−1^. Gal-UA was highest in Group D at 19.49 µg·mg^−1^. The highest molar content of Gal was observed in Group C at 10.12 µg·mg^−1^. Ara was the least abundant, detected only in Groups C and D at 2.91 µg/mg and 2.60 µg·mg^−1^, respectively. The total monosaccharide content ranged from 620.47 µg·mg^−1^ to 683.98 µg·mg^−1^, with Group D showing the highest total monosaccharide content.

### 3.3. Differential Analysis of Metabolites in GE

#### 3.3.1. Metabolite Classification and Cluster Analysis

**Figure 2:**
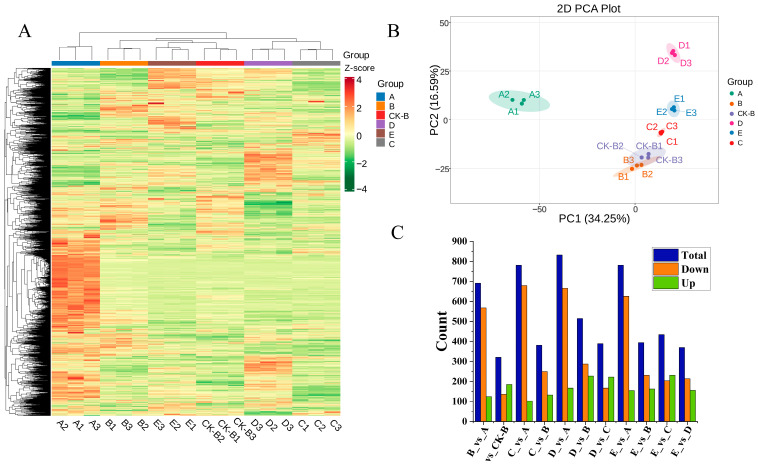
Classification and cluster analysis of Gastrodia elata metabolites. (**A**) Heatmap of metabolite expression levels across all groups, (**B**) PCA plot, (**C**) number of differentially regulated metabolites. A–E: Five groups of *Gastrodia elata* cultivated with *Armillaria mellea* from different sources. CK-B: The control of Group B (cultivated in different regions).

To further compare the metabolic characteristics of GE symbiotic with different sources of *A. mellea*, this study conducted a broad-spectrum targeted metabolomic analysis using the UPLC-MS/MS platform. A total of 2418 metabolites were detected and classified into 13 categories based on their chemical structures (Figure 2A). Among them, 429 metabolites belonged to the “other” category, accounting for 17.74% of the total; 377 were amino acids and their derivatives (15.59%); 287 were alkaloids and 287 were lipids, each representing 11.87%; 255 were terpenoids (10.55%); 252 were phenolic acids (10.42%); 191 were flavonoids (7.90%); 122 were organic acids (5.55%); 89 were nucleotides and their derivatives (3.68%); 78 were lignans/coumarins (3.23%); 40 were quinones (1.65%); 9 were steroids (0.37%); and 2 were tannins (0.08%). This quantitative distribution provides a comprehensive metabolic baseline for subsequent differential metabolite screening and functional enrichment analysis.

To visually evaluate metabolic differences across treatment groups, we conducted PCA on all samples (Figure 2B). The PCA results demonstrated tight clustering among biological replicates, confirming strong experimental reproducibility and data reliability. The first two principal components (PC1 and PC2) accounted for 34.25% and 16.59% of the total variance, respectively. Based on the score distribution of PC1 and PC2, the samples could be clearly divided into six clusters, suggesting significant differences in metabolic profiles among the groups. It is noteworthy that there is an overlap between treatment group B and its control group B-CK in the two-dimensional space, indicating a relatively small difference between the two. Group A exhibited substantial metabolic differences compared to the other groups.

#### 3.3.2. Differential Metabolite Analysis

To screen for significantly differential metabolites, this study set thresholds of VIP ≥ 1.0, a fold-change ≥ 2, and ≤0.5 (Figure 2C). The number of differential metabolites compared with group A as the control was as follows: B vs. A, 691 differential metabolites in total (124 up and 567 down); C vs. A, 781 differential metabolites (102 up and 679 down); D vs. A, 832 differential metabolites (167 up and 665 down); E vs. A, 781 differential metabolites (155 up and 626 down). The comparison between B and its own control group CK-B, cultivated in different plots, revealed the following: B vs. CK-B, 321 differential metabolites (185 up and 136 down). This indicates that samples with similar climatic and geographical conditions had a minor impact on the quality of GE, suggesting that the differences in *A. mellea* were the primary factor driving metabolic variations in GE in this study. The results of other pairwise comparisons are as shown in Figure 2C.

**Figure 3:**
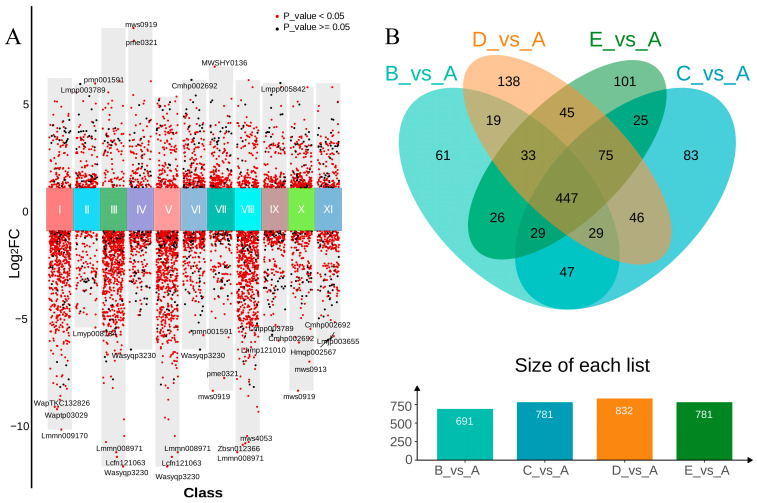
Distribution and intersection diagram of differential metabolites. (**A**): volcano plot of differential metabolites; (**B**): venn intersection plot of differential metabolites; I: B_vs_A, II: B_vs_CK-B, III: C_vs_A, IV: C_vs_B, V: D_vs_A, VI: D_vs_B, VII: D_vs_C, VIII: E_vs_A, IX: E_vs_B, X: E_vs_C, XI: E_vs_D; A–E: five groups of *Gastrodia elata* cultivated with *Armillaria mellea* from different sources.

The above statistics reveal that the metabolic differences between Group A and the other four groups are the most significant. We further generated a volcano plot (red dots represent significant metabolites with *p* < 0.05 and meeting the fold-change criteria, while black dots denote non-significant metabolites). The plot clearly demonstrates that Group A exhibits a markedly distinct metabolic profile compared to the other groups (Figure 3A). Subsequently, a Venn diagram analysis was performed on the differential metabolites obtained from comparisons with Group A (Figure 3B), with results as follows: 447 differential metabolites were shared across all four comparison groups (B vs. A, C vs. A, D vs. A, and E vs. A). Additionally, 61 metabolites were unique to B vs. A, 83 to C vs. A, 61 to D vs. A, and 101 to E vs. A. The intersections and unique metabolites are visually presented in the Venn diagram, further illustrating the pronounced metabolic distinctions of Group A relative to the other groups.

### 3.4. Transcriptome Data Analysis of GE

#### 3.4.1. Transcriptome Sequencing Analysis of GE

To elucidate the molecular mechanisms underlying differences in metabolite accumulation among GE symbiotic with different *A. mellea* sources, this study conducted whole-transcriptome sequencing on samples of GE from five *A. mellea* sources. A total of 1,037,551,196 raw reads were obtained, and after removing low-quality sequences, 964,110,904 clean reads were retained. The sequencing quality metrics showed Q30 values ranging from 95.84% to 96.78%, and GC content between 46.46% and 49.10%, indicating reliable data quality suitable for subsequent analysis.

**Figure 4:**
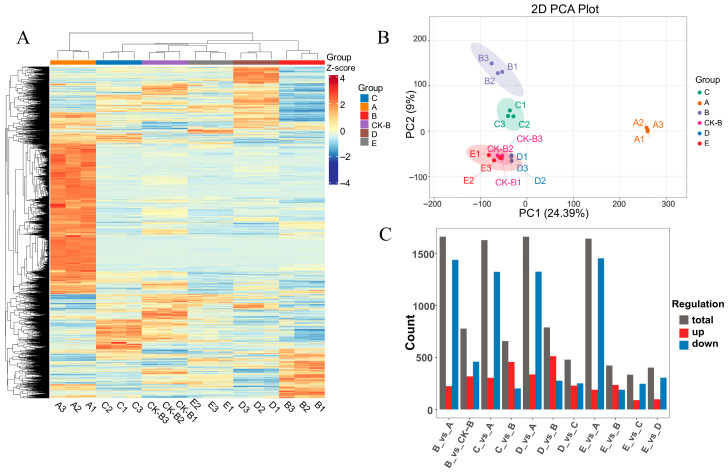
Distribution of *Gastrodia elata* genes in each group and clustering between groups. (**A**) Heatmap of gene expression levels across all groups, (**B**) PCA plot, (**C**) number of differentially regulated genes; A–E: five groups of *Gastrodia elata* cultivated with *Armillaria mellea* from different sources. CK-B: the control of Group B (cultivated in different regions).

To visually assess transcriptional differences among the samples, principal component analysis (PCA) was performed on all samples. The PCA score plot (Figure 4B) classified the samples into six groups, with the PC1 and PC2 explaining 24.39% and 9.00% of the total variance, respectively. This further confirmed the overall transcriptional differences among the samples.

#### 3.4.2. Differential Gene Analysis

In this study’s gene annotation library, functional annotations were completed for 14,637 genes. Differentially expressed genes (DEGs) between groups were then identified using the thresholds |Log2FC| ≥ 1 and FDR < 0.05 (*p* < 0.05). The results of each experimental group compared to control group A are as follows (Figure 4C): B vs. A, 1659 DEGs (222 upregulated, 1437 downregulated); C vs. A, 1625 DEGs (304 upregulated, 1321 downregulated); D vs. A, 1659 DEGs (336 upregulated, 1323 downregulated); E vs. A, 1640 DEGs (188 upregulated, 1452 downregulated). Additionally, the comparison between group B and its control group CK-B revealed 776 DEGs (318 upregulated, 458 downregulated), suggesting relatively minor gene expression differences under similar climatic and geographical conditions. The remaining pairwise comparisons are detailed in Figure 4C.

**Figure 5:**
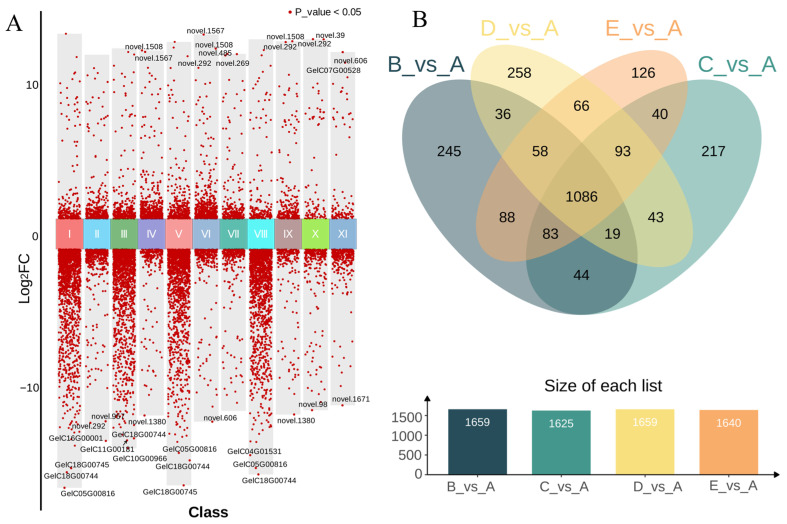
Volcano plot of DEGs (**A**) and Venn intersection plot (**B**). I: B_vs_A, II: B_vs_CK-B, III: C_vs_A, IV: C_vs_B, V: D_vs_A, VI: D_vs_B, VII: D_vs_C, VIII: E_vs_A, IX: E_vs_B, X: E_vs_C, XI: E_vs_D; A–E: five groups of *Gastrodia elata* cultivated with *Armillaria mellea* from different sources.

The differential gene clustering heatmap (Figure 4A) and volcano plot (Figure 5A) reveal significant differences in gene expression levels between Group A and the other four groups. The clustering results distinctly separate Group A, and the abundance of red dots in the volcano plot further confirms this observation. Subsequently, Venn intersection analysis was performed on all differentially expressed genes (DEGs) compared with Group A (Figure 5B), yielding the following results: 1086 DEGs were common across all four comparisons (B vs. A, C vs. A, D vs. A, E vs. A). Additionally, 245 DEGs were unique to B vs. A, 217 to C vs. A, 258 to D vs. A, and 126 to E vs. A. The distribution of these shared and unique genes indicates that while there are common differential responses among the groups, each group also exhibits distinct gene expression patterns. These findings provide critical insights for subsequent functional enrichment analysis and the construction of metabolic regulatory networks.

### 3.5. Integrated Analysis of DEMs and DEGs

**Figure 6:**
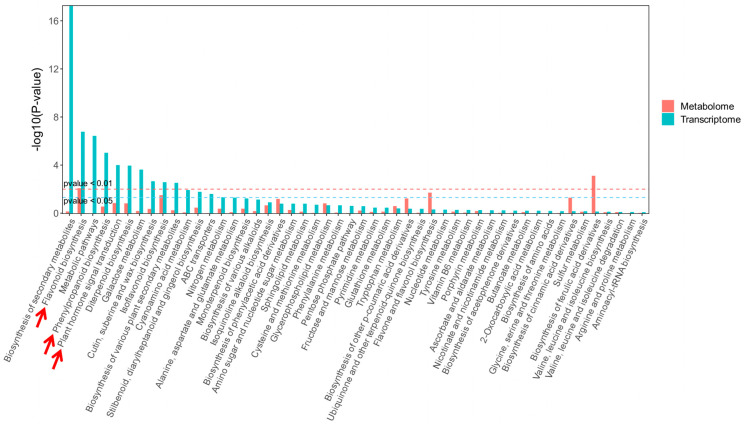
Enrichment results of differentially expressed genes and differential metabolites.

**Figure 7:**
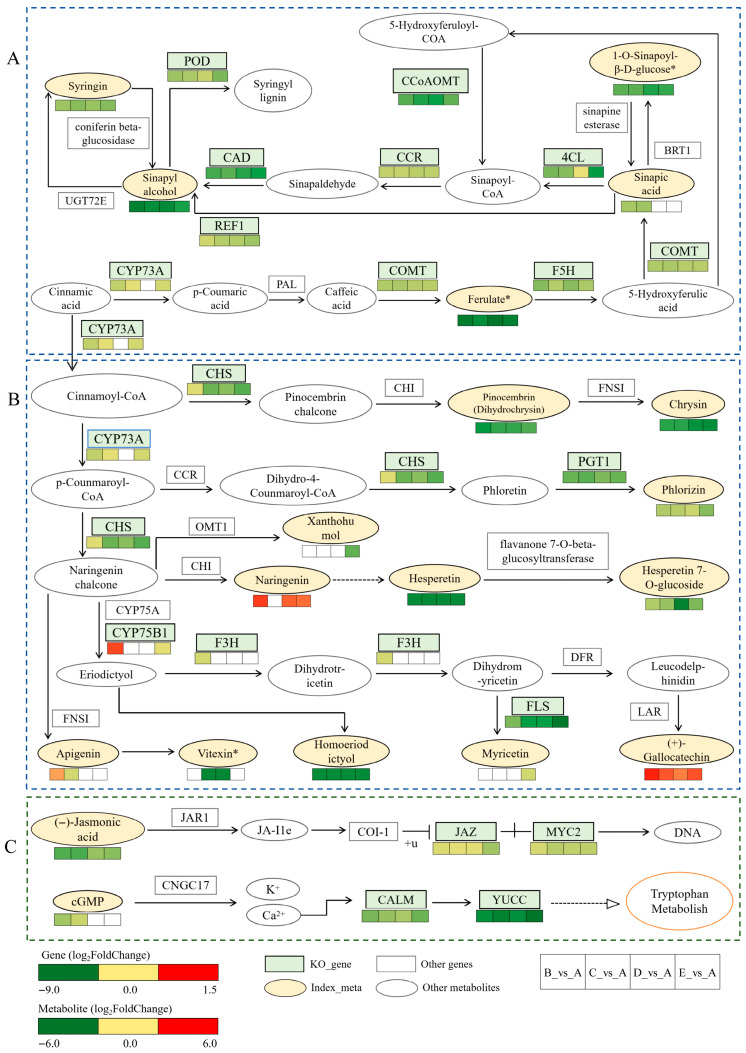
Heatmap of KEGG pathways for differentially expressed genes and differential metabolites. (**A**) Phenylpropanoid biosynthesis (ko00940), (**B**) Flavonoid biosynthesis (ko00941), (**C**) Plant hormone signal transduction (ko04075). White in the significance box indicates that the group was not annotated in the pathway.

Based on Pearson correlation analysis, 1433 DEGs showed significant positive/negative correlations with 665 DEMs. KEGG annotation was performed for all DEGs and DEMs, followed by a comprehensive analysis of their enrichment results (Figure 6). The integrated KEGG enrichment analysis revealed that the four comparison groups (B vs. A, C vs. A, D vs. A, E vs. A) shared three major enriched pathways: Flavonoid biosynthesis (ko00941), Phenylpropanoid biosynthesis (ko00940), and Plant hormone signal transduction (ko04075). Three pathway diagrams are shown in Supplementary Materials (Figures S4–S6). The regulatory relationships between DEGs and DEMs in these three pathways were visualized using pathway heatmaps (Figure 7). In the heatmaps, upregulated (red) or downregulated (green) gene expression exhibited consistent or opposite trends with the corresponding changes in metabolite abundance. These analyses indirectly suggest that the source of *A. mellea* may lead to variations in GE metabolites by regulating related biosynthesis pathways, but further validation through fungal omics studies is required.

**Figure 8:**
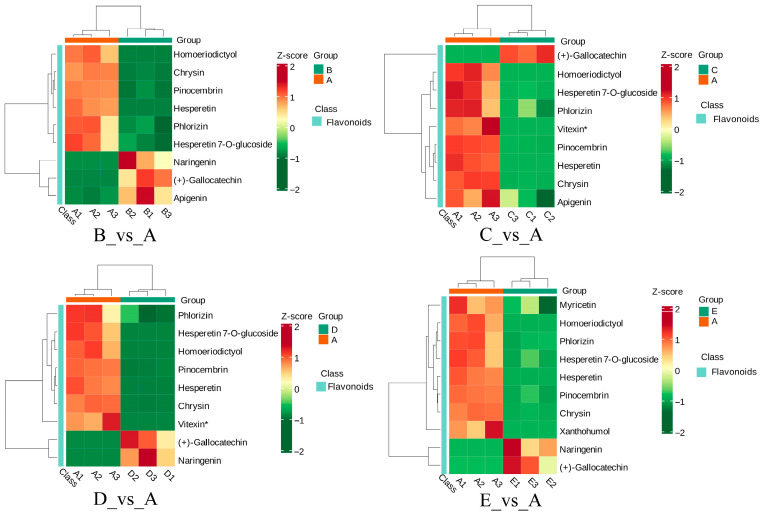
Expression levels of metabolites involved in the flavonoid biosynthesis pathway. A–E: Five groups of *Gastrodia elata* cultivated with *Armillaria mellea* from different sources.

We can observe that in the heatmap of ko00941, all commonly enriched DEMs in the comparison groups using A as the control showed downregulated expression (Figure 7B). Among them, Naringenin appeared only in the B_vs_A, D_vs_A, and E_vs_A groups, while Apigenin was found only in the B_vs_A and C_vs_A groups. The expression trends of metabolites in each comparison group are summarized as follows: B_vs_A, Naringenin, Apigenin, and (+)-Gallocatechin were downregulated, while the other metabolites were upregulated; C_vs_A, all metabolites were upregulated except for (+)-Gallocatechin, which was downregulated; D_vs_A and E_vs_A, Naringenin and (+)-Gallocatechin were downregulated, while the remaining metabolites were upregulated (Figure 8). In the same heatmap, the expression trends of DEGs are also clearly visible: CHS, PGT1, and FLS were downregulated in all comparison groups. F3H was downregulated only in B_vs_A. CYP73A was downregulated in B_vs_A, C_vs_A, and E_vs_A. CYP75B1 was upregulated in B_vs_A but downregulated in E_vs_A. Overall, most flavonoid metabolites and key synthesis genes in groups B, C, and D exhibited a downregulation trend, while metabolites and genes in group A were generally upregulated. This may facilitate the synthesis and accumulation of flavonoids.

The heatmap of ko00940 (Figure 7A) shows that four co-enriched DEMs—1-O-Sinapoyl-β-D-glucose*, Ferulate*, Syringin, and Sinapyl alcohol—were downregulated in all comparison groups. Sinapate appeared only in B_vs_A and C_vs_A and was also downregulated. All key co-enriched genes/enzymes in the pathway were downregulated, including the following: 4CL (GelC05G00275, GelC04G00359), COMT (GelC03G01028), REF1 (GelC06G01136), CCR (GelC04G01145, GelC15G00275), F5H (GelC15G00173, GelC02G01836), CCoAOMT (GelC01G00401, GelC05G01100), POD (GelC04G00296, GelC01G00462, GelC13G00689), and CAD (GelC09G00591, GelC01G01650). Among these, CYP73A was not detected in D_vs_A. Although the heatmap indicates an overall downregulation trend for metabolites and genes in groups B, C, and D, the differential metabolites and genes in group A were relatively upregulated (compared to other groups), which may contribute to the synthesis and accumulation of phenylpropanoid metabolites.

The heatmap of ko04075 (Figure 7C) revealed that the only commonly enriched DEMs, (−)-Jasmonic acid (JA), were significantly downregulated in all comparison groups. 3′,5′-Cyclic GMP (cGMP) appeared only in B_vs_A and C_vs_A and was also downregulated. The DEGs in this pathway were all downregulated, including the following: JAZ (GelC15G00723), MYC2 (GelC02G01547, GelC13G00338), CALM (GelC05G00386, GelC15G00684, GelC16G00500, GelC02G00978), and YUCCA (GelC05G00629). These DEMs and DEGs may influence the transduction of plant hormone signaling.

### 3.6. Expression Correlation Analysis

**Figure 9:**
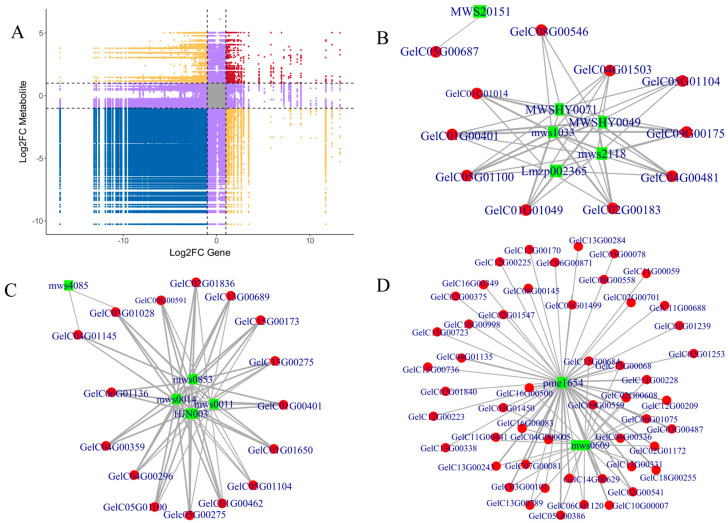
Correlation analysis of differentially expressed genes and differentially metabolites. (**A**) Nine-quadrant plot, (**B**–**D**) correlation network diagrams of ko00941, ko00940, and ko04075, green dots represent compounds, and red dots represent genes.

To visually illustrate the association between gene expression and metabolite abundance within the same biological sample, we first screened gene–metabolites that met the criteria of a Pearson correlation coefficient |r| > 0.8 and *p* < 0.05 (remaining significant after FDR correction). Subsequently, a nine-quadrant plot was constructed using the fold change as the coordinate (Figure 9A), taking B vs. A as an example, where the horizontal and vertical coordinates represent the fold-change (Log2FC) of genes and metabolites, respectively. Two dashed lines parallel to the axes correspond to the threshold of |Log2FC| ≥ 1 (i.e., a ≥2-fold change in gene or metabolite expression), dividing the plot into nine quadrants (1–9) from left to right and top to bottom. The darker and larger the points in each quadrant, the stronger the correlation. In this study, the gene–metabolite pairs co-enriched in the three KEGG pathways (ko04075, ko00940, and ko00941) were predominantly located in Quadrant 7, with darker/larger points, indicating their |r| values were close to 1 and all met |Log2FC| ≥ 1. Thus, the genes in these pathways were synchronously downregulated with their corresponding metabolites, exhibiting a positive expression pattern—i.e., reduced gene expression may directly drive decreased metabolite abundance. In contrast, pairs in Quadrant 1 (e.g., (+)-Gallocatechin) showed downregulated genes but upregulated metabolites, suggesting potential negative regulation, metabolic feedback inhibition, or other regulatory mechanisms, warranting further experimental validation. Overall, the regulatory directions of gene–metabolite interactions displayed in the nine-quadrant plot align with the pathway heatmaps, further validating the reliability of the threshold settings and visualization methods.

In the pairs satisfying the Pearson correlation coefficient |r| > 0.8, the correlation network diagram (taking B_vs_A as an example, Figure 9) revealed a strong metabolite–gene association structure. In the diagram, red represents genes, green represents metabolites, and solid lines indicate positive correlations. The ko00941 network diagram (Figure 9B) shows that metabolites such as chrysin, hesperetin, homoeriodictyol, phlorizin, and hesperetin-7-O-glucoside form a tightly interconnected positive correlation network with the CHS, PGT1, and FLS genes. Notably, apigenin exhibited a strong association only with CYP75B1 (GelC05G00687), likely suggesting the unique role of this enzyme in a specific branch pathway. Some metabolites also showed high correlations with caffeoyl-CoA O-methyltransferase (GelC05G01104, GelC05G01100), further indicating the auxiliary role of this enzyme in flavonoid synthesis.

In the ko00940 network diagram (Figure 9C), Sinapyl alcohol, Ferulate*, Syringin, 1-O-Sinapoyl-β-D-glucose*, and Sinapate all form a positively correlated network centered around key enzymes such as cinnamyl-alcohol dehydrogenase, F5H, 4CL, and CCR, constituting the backbone of phenylpropanoid metabolism. Sinapate forms a unique association pair with COMT (GelC03G01028) and CCR (GelC04G01145), potentially exhibiting specificity in phenolic side chain modification.

The network correlation analysis of ko04075 (Figure 9D) revealed that (−)-Jasmonic acid is highly correlated with JAZ and MYC2 genes, suggesting that JA signaling may primarily be transmitted through these two types of transcription factors. Meanwhile, 3′,5′-Cyclic GMP showed strong associations with CALM and YUCCA, indicating its potential regulatory role in calcium signaling and tryptophan metabolism. The correlations of both compounds align with the overall downregulation trend observed in the heatmap.

## 4. Discussion

The widely recognized “Growth-Defense Trade-off” (GDT) theory in botany posits that due to limited resources available to plants (such as carbon, nitrogen, and phosphorus), they must allocate resources meticulously between two core biological activities: “growth” (e.g., increasing biomass) and “defense” (e.g., synthesizing chemical compounds). During the growth of GE, when *A. mellea* hyphae invade its cortex, the plant secretes enzymes to break down polysaccharides and proteins in the hyphae, converting them into carbon and nitrogen sources needed for its own growth [36]. In this study, Group A exhibited significantly higher accumulation of GAS, HBA, and parishin metabolites compared to other groups, although its yield was lower than that of Group B. Conversely, Group B achieved the highest yield but had lower concentrations of active compounds. This pattern aligns closely with the GDT theory. Group A likely prioritized defense over growth by downregulating genes involved in growth and increasing the secretion of defense proteins, directing carbon and nitrogen resources toward the production of defensive compounds [37]. This shift may explain Group A’s superior performance in active compound accumulation. Additionally, Group A’s polysaccharides had a relatively smaller molecular weight and the highest glucose (Glc) content, suggesting a simpler polysaccharide structure that could potentially influence medicinal properties differently.

An integrated analysis of metabolomics and transcriptomics from GE cultivated with *A. mellea* from different sources revealed key pathways co-enriched with differentially expressed genes and metabolites. In the ko00941 pathway, all differential metabolites in Group A showed upregulation except for (+)-Gallocatechin. Genes such as CHS, PGT1, and FLS, located upstream of metabolites like chrysin, hesperetin, and phlorizin, serve as critical rate-limiting steps in flavonoid biosynthesis. These genes were upregulated in Group A, likely contributing to its advantage in flavonoid biosynthesis and leading to significantly elevated levels of most co-differential metabolites. Although (+)-Gallocatechin was downregulated in Group A, its upstream genes (CHS, PGT1, FLS) exhibited transcriptional upregulation. This phenomenon may reflect a cellular negative feedback mechanism: once (+)-Gallocatechin reaches a certain concentration, cells may suppress the expression of upstream enzyme genes to prevent excessive accumulation of flavonoids. Previous studies have also demonstrated that the downregulation of CHS can coordinately regulate flavonoid biosynthesis, playing a crucial negative regulatory role [38]. This negative feedback was further validated by experiments showing that metabolite synthesis could be restored through substrate feeding even after CHS gene silencing, indicating that transcriptional inhibition responds to metabolite levels [39].

The phenylpropanoid metabolism provides precursors for flavonoid synthesis, and CHS, as the first enzyme in flavonoid biosynthesis, can channel carbon flow from the phenylpropanoid pathway into flavonoid production. The coordinated regulation of these two pathways further highlights the advantage of Group A in promoting the synthesis of flavonoid metabolites. Key genes such as 4CL, COMT, and POD play critical roles in phenylpropanoid metabolism and plant defense [40,41]. In the ko00940 pathway, these genes exhibit higher expression levels in Group A. 4CL converts phenylalanine into p-coumaroyl-CoA, serving as a bridge connecting phenylpropanoid metabolism with flavonoid synthesis. Environmental stimuli (e.g., light, temperature) can activate 4CL, enhancing carbon flow toward flavonoid synthesis and thereby increasing the metabolite yield [42]. Meanwhile, COMT and POD participate in subsequent hydroxylation and oxidation reactions, further driving the accumulation of flavonoids and their derivatives.

The synthesis of active components such as GAS, HBA, and parishin in GE may be co-regulated by the phenylpropanoid metabolic pathway and flavonoid biosynthesis pathway [43]. Phenylalanine is converted into GAS and HBA via the phenylpropanoid pathway, which subsequently forms parishin through the polymerization of three GAS molecules and one citric acid molecule [44]. When the flavonoid biosynthesis pathway is inhibited, the diversion of phenylalanine toward flavonoids weakens, potentially leading to an insufficient supply of downstream substrates like GAS, resulting in decreased GAS levels in groups B, C, D, and E. Glucose not only serves as a structural unit but also acts as a signaling molecule involved in metabolic networks, closely linked to the phenylpropanoid pathway [45]. A previous whole-genome analysis of Armillaria gallica Jzi34 revealed that glycosyltransferase (GT) genes are the most abundant [46], which may also explain the regulatory differences in polysaccharide synthesis in GE by different *A. mellea*. Jasmonic acid (JA) can upregulate the expression of secondary metabolic genes by activating defense-related genes such as PAL and CHS [47]. Additionally, studies have shown that JA, as a stress signal, can simultaneously activate primary and secondary metabolism, leading to a significant increase in phenolic compounds [48,49]. This suggests that the increase in JA levels may be associated with the accumulation of phenolic compounds such as GAS, HBA, p-hydroxybenzaldehyde, and gastrodin. This assertion is a moderate speculation and requires further experimental validation in the future.

This study reveals the association between the origin of *A. mellea* and the metabolic and transcriptional responses of GE, suggesting that high-quality strains may regulate host metabolic pathways by secreting specific signaling molecules, influencing secondary metabolite accumulation [50]. However, this research lacks direct analysis of the *A. mellea* genome and its secreted metabolites, failing to clarify whether the strains exhibit genetic or geographical differences. The conclusions are host (GE)-centric and indirectly inferred, representing a limitation. Future research will incorporate fungal omics analysis for deeper investigation.

## 5. Conclusions

This study reveals differences in the metabolic profiles and gene expression of GE when cultivated with *A. mellea* from different sources. Indirect deduction centered on the host (GE) suggests that *A. mellea* A may induce the upregulation of numerous metabolites and genes in GE, thereby promoting the accumulation of active components, while *A. mellea* B appears more conducive to enhancing the yield. Systematic analysis of co-enriched pathways, including flavonoids, phenylpropanoids, and plant hormone signaling, identified several key metabolites and regulatory genes in GE. Subsequent research will focus on validating these key genes and conducting in-depth studies combined with fungal omics analysis, aiming to provide a molecular basis for constructing efficient “*A. mellea*-GE” combinations.

## Figures and Tables

**Table 1:** Polysaccharide extraction rate and monosaccharide composition content.

Group *	Polysaccharide Extraction Rate (%)	Monosaccharide Content (µg·mg^−1^)				
		Ara	Gal	Glc	Gal-UA	Total
A	7.33	0.00	4.17	668.08	9.28	681.53
B	4.00	0.00	4.30	664.45	7.84	676.60
C	5.00	2.91	8.98	590.57	18.01	620.47
D	10.66	2.60	10.12	651.77	19.49	683.98
E	10.66	0.00	4.58	667.54	5.55	677.67

* A–E: Five groups of *Gastrodia elata* cultivated with *Armillaria mellea* from different sources.

**Table 2:** Molecular weight distribution parameters of polysaccharides from *Gastrodia elata* cultivated with *Armillaria mellea* from different sources *.

Sample	Time (min)	Mn (g·mol^−1^)	Mw (g·mol^−1^)	Proportion	PDI
A	5.114	>1,000,000	>1,000,000	34.01%	-
	9.292	12,091	22,235	35.56%	1.84
	10.600	866	1273	30.44%	1.47
B	6.534	87,128	1,951,769	100.00%	22.40
C	5.704	284,616	935,643	52.39%	3.29
D	5.667	72,441	2,520,299	100.00%	34.79
E	5.717	1,523,219	3,863,769	5.13%	2.54
	9.216	12,767	24,410	50.39%	1.91
	10.589	851	1272	44.49%	1.49

* Mn: number-average molecular weight, Mw: weight-average molecular weight, PDI (polydispersity index) = Mw/Mn; A–E: five groups of *Gastrodia elata* cultivated with *Armillaria mellea* from different sources.

## Data Availability

All data are available in the manuscript and Supplementary Materials. The transcriptome datasets were submitted to a public repository (NCBI SRA). Accession numbers range from SAMN54581423 to SAMN54581440.

## Supplementary Materials

The following supporting information can be downloaded at: https://www.mdpi.com/article/10.3390/biology15020196/s1, Figure S1: The process of cultivating *Gastrodia elata* with Armillaria from different sources and a brief diagram of field design; Figure S2: The molecular weight distribution profile of *Gastrodia elata* polysaccharides was determined via gel filtration chromatography; Figure S3: The monosaccharide TIC chromatograms of sample (A) and reference standard (B); Figure S4: Flavonoid biosynthesis pathway (ko00941); Figure S5: Phenylpropanoid biosynthesis pathway (ko00940); Figure S6: Plant hormone signal transduction pathway (ko04075); Table S1: *Armillaria mellea* source information; Table S2: Cultivation of fungal material and methods for growing *Gastrodia elata*; Table S3: Standard curve of linear regression for main active components of GE; Table S4: Monosaccharide component regression curve parameters. Table S5: Content of main active ingredients (mg/g) in different groups of *Gastrodia elata* (*n* = 3); metabolome_sample_data.xlsx.
