# Supplementary material for: Comprehensive Metabolomic–Transcriptomic Analysis of the Regulatory Effects of Armillaria mellea Source Differences on Secondary Metabolism in Gastrodia elata
Source: Biology (Basel). 2026 Jan 21;15(2):196. doi: 10.3390/biology15020196 (PMC12837748; doi:10.3390/biology15020196)
Supplement: Supplementary file 1 [file biology-15-00196-s001.zip › biology-4091558-supplementary.pdf]

Supplementary Materials

FIGURES

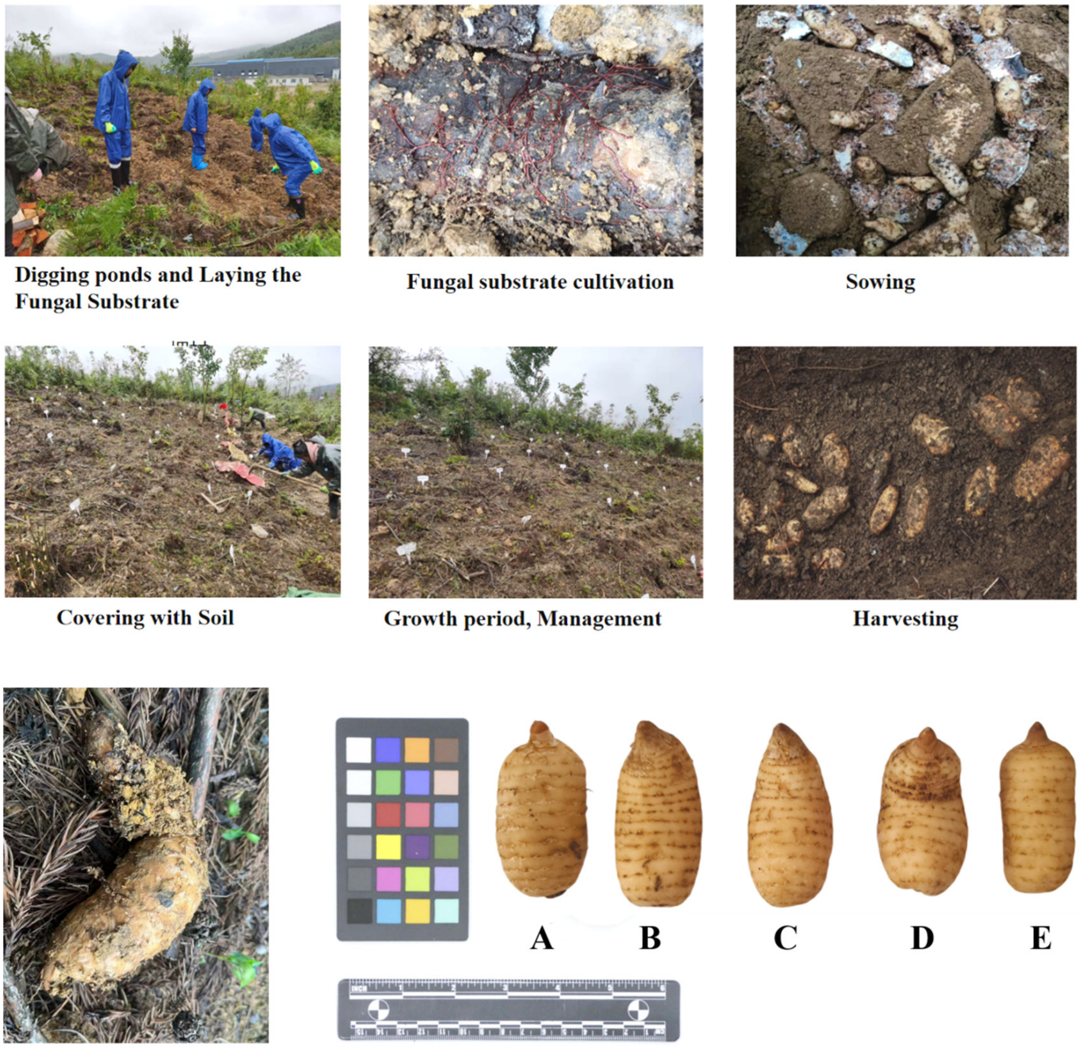

Figure S1 The process of cultivating Gastrodia elata with Armillaria mellea from different sources

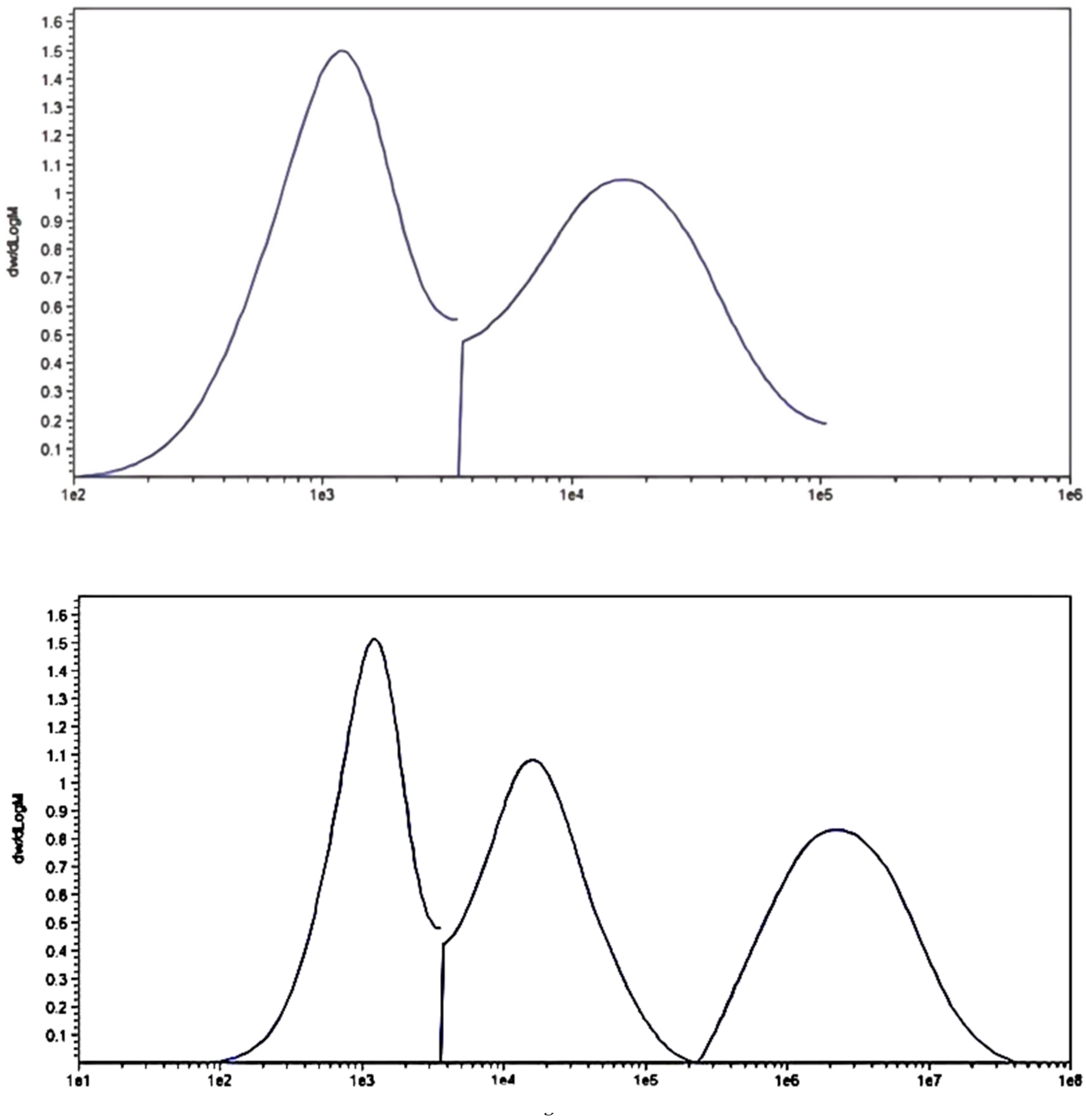

Figure S2 The molecular weight distribution profile of *Gastrodia elata* polysaccharides was determined by gel filtration chromatography

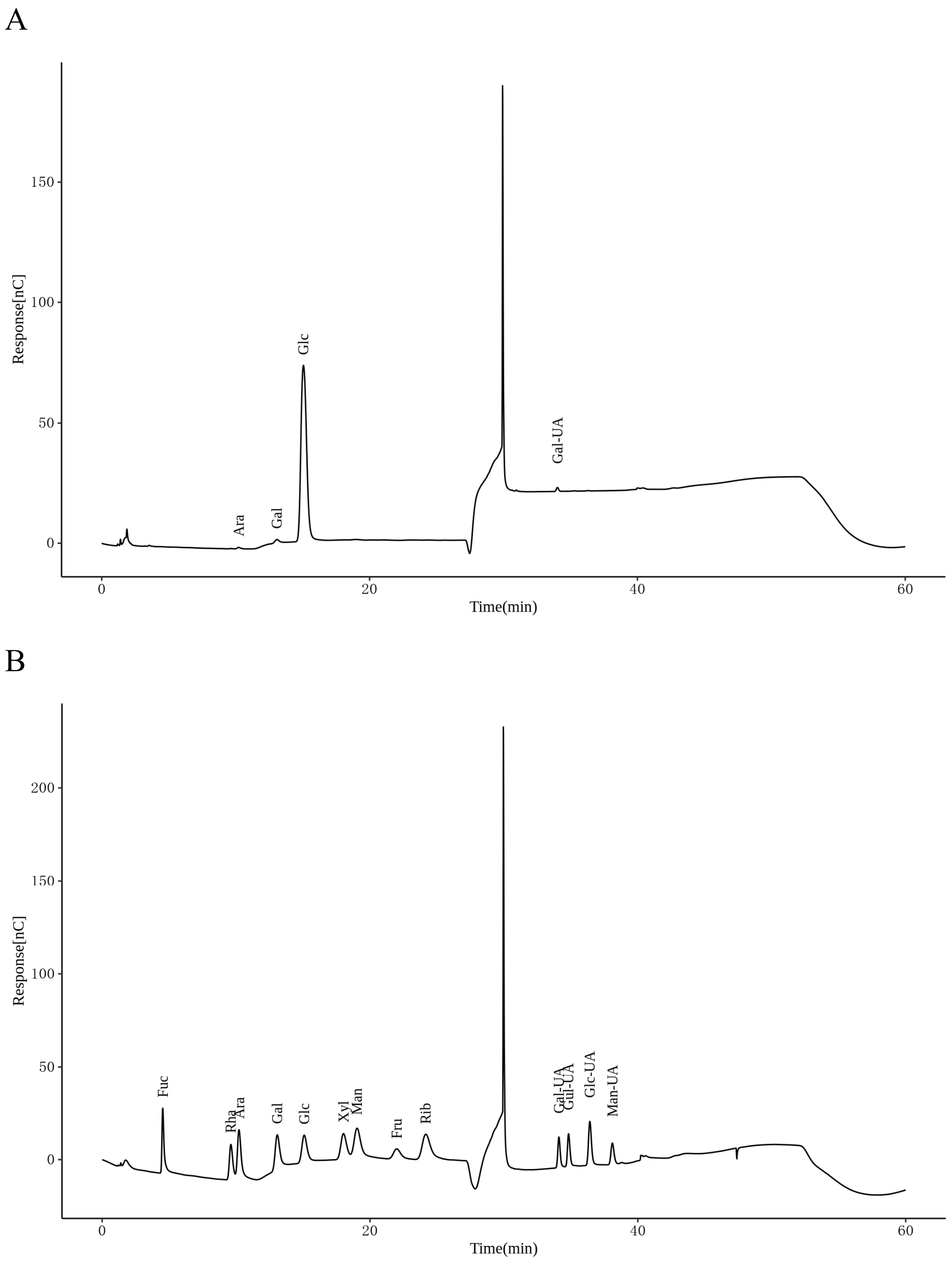

Figure S3 The monosaccharide TIC chromatograms of sample (A) and reference standard (B)

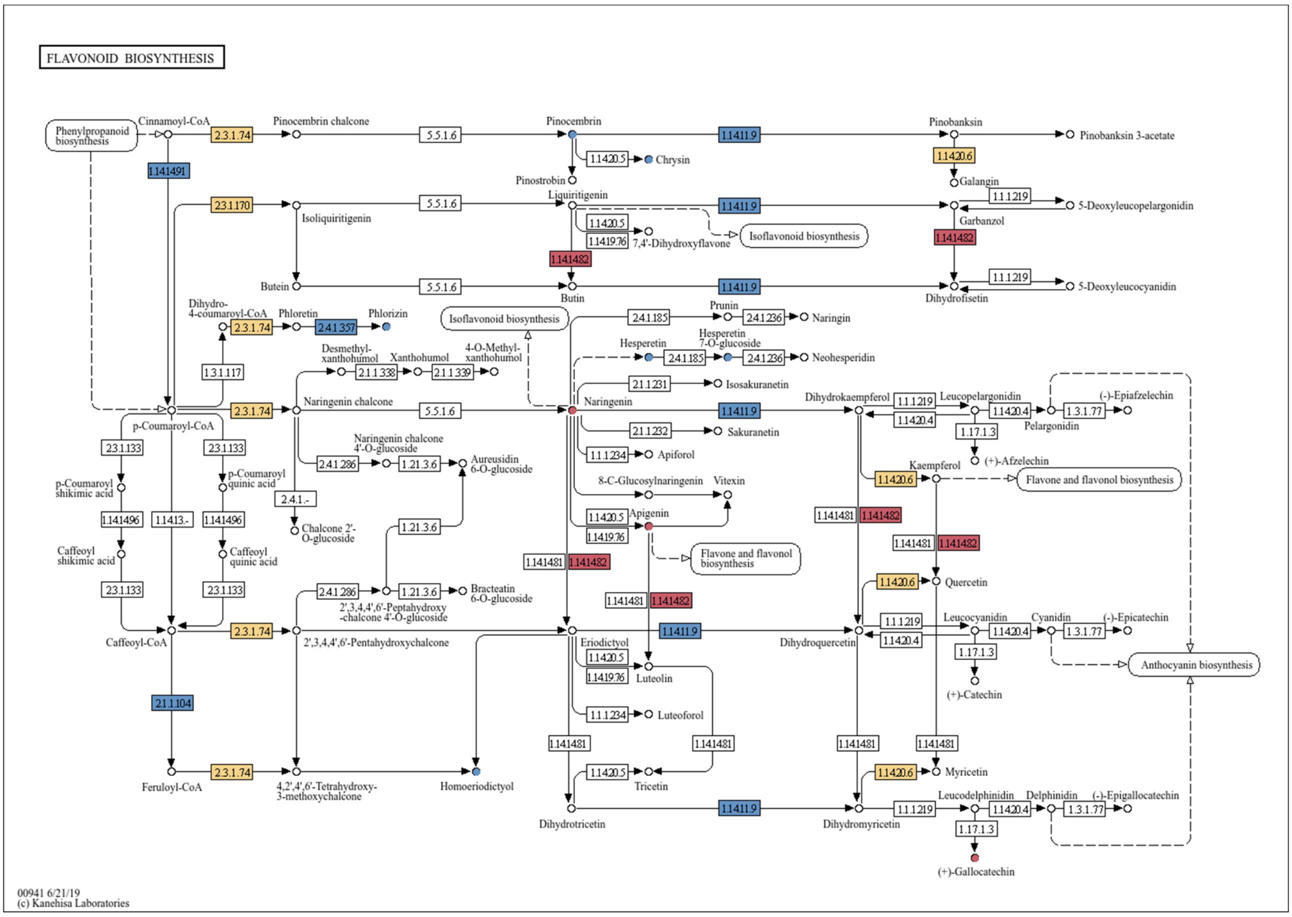

Figure S4 Flavonoid biosynthesis pathway (ko00941)

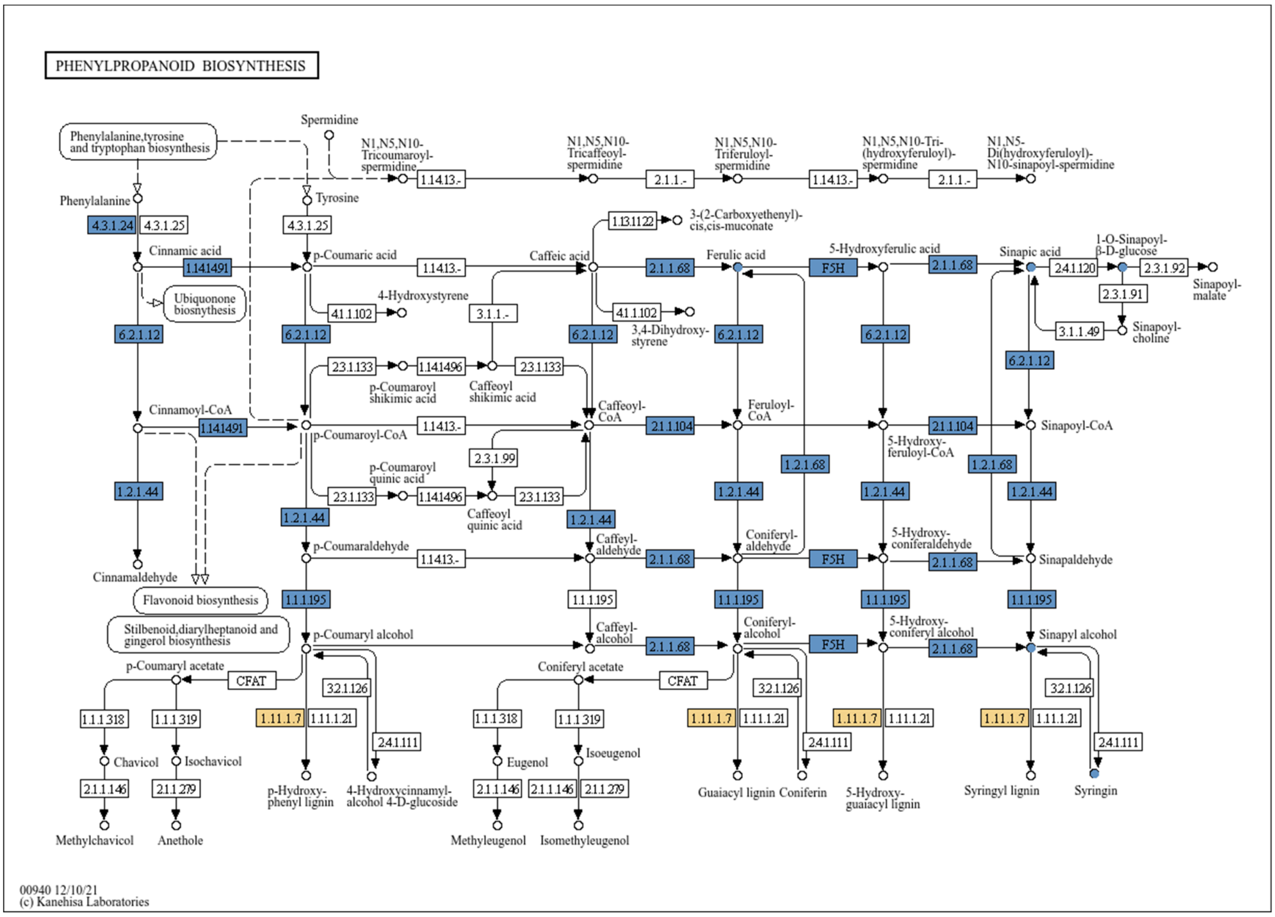

Figure S5 Phenylpropanoid biosynthesis pathway (ko00940)

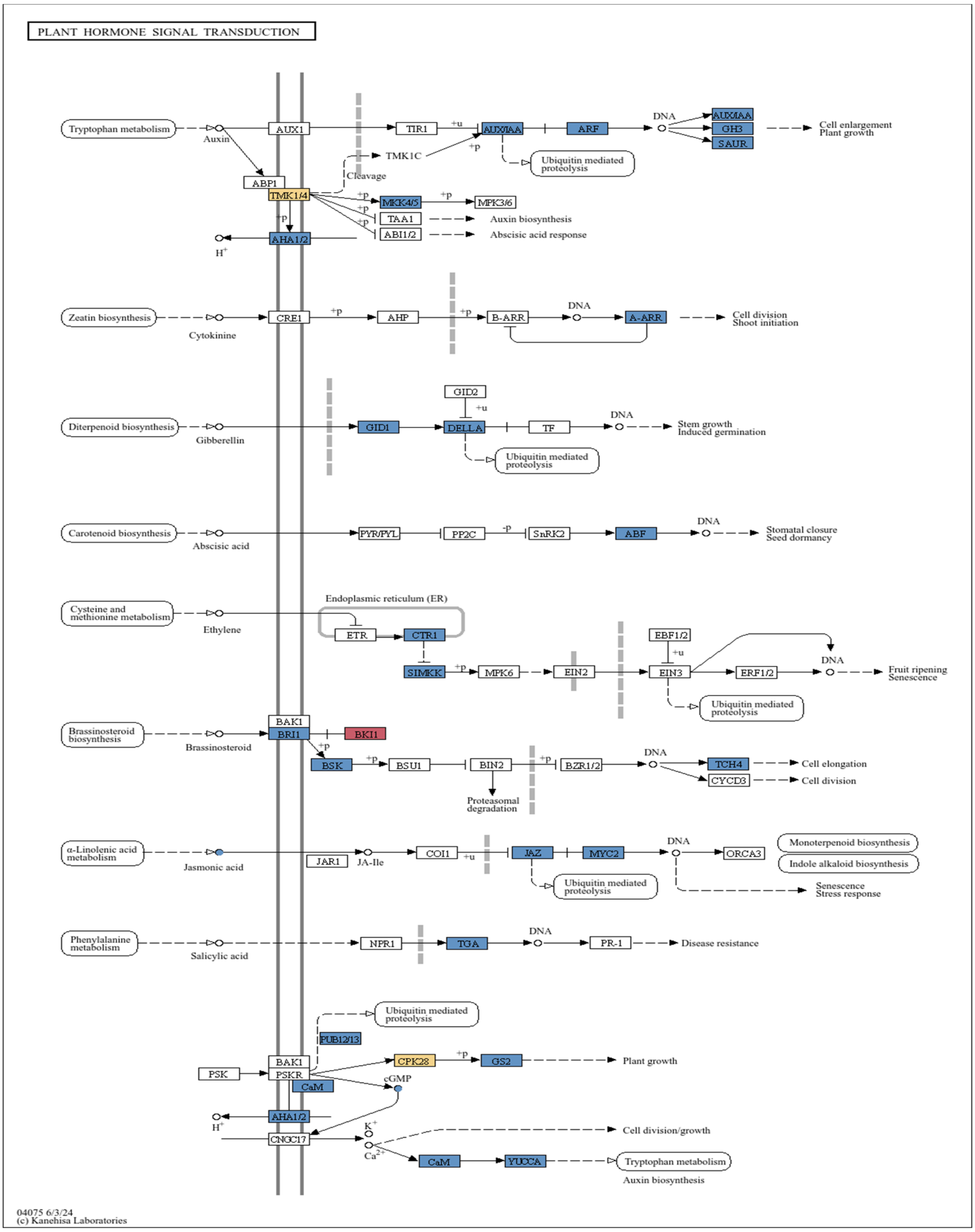

Figure S6 Plant hormone signal transduction pathway (ko04075)

TABLES

Table S1 Armillaria mellea source information

Number of Armillaria mellea	Number of Gastrodia elata	Source of Armillaria mellea	culture medium
A	Group A	Shaanxi Sensheng Fungal Industry Technology Co., Ltd.	Branches
B	Group B	Yichang Hongsheng Biotechnology Co., Ltd.	Corn kernels
C	Group C	Yongqian Fungal Spawn Farm, Tuohe Village, Yuele Town, Daguan County	Branches
D	Group D	Zhaotong Gastrodia Research Institute	Branches
E	Group E	Yunnan Senhao Fungal Industry Co., Ltd.	Seed husks

Table S2 Cultivation of fungal material and methods for growing Gastrodia elata

Processing	Description of the cultivation process and specification size	
Fungal Material Cultivation (September 2022)	1. Digging pits	Length× width × height is: 100 cm × 50 cm × 20 cm, with a spacing of 50 cm between two holes. Each treatment setting consists of 5 pits.
	2.Laying the Fungal Substrate	The fungal substrate is Quercus glauca, with a diameter of 5-10 cm and a length of 20-30 cm. Each pit requires 15 kg.
	3.Placing Armillaria mellea	Use 1 bottle (800 g) of Armillaria mellea per pit. Break the Armillaria mellea into thumb-sized pieces and place the pieces closely against the fungal substrate.
	4.Covering with Soil	Apply a layer of soil approximately 5-10 cm thick, ensuring the covered height is slightly above the surrounding ground level.
Sowing and Management (January 2023 to January 2024)	In January 2024, seed of Gastrodia elata (20 pieces, approximately 200 g) are to be placed in the fungal pits, covered with soil, and conduct unified management and monitoring.	
Harvesting (January 2024)	The planting cycle is one year, starting from the growth of Gastrodia elata seeds to mature Gastrodia elata arrows, with a unified harvest scheduled for January 2024.	

Table S3 Standard curve of linear regression for main active components of GE

Reference standard	Regression equation	R^2
GAS	Y = 1411.32X + 1636.84	0.9999
HBA	Y = 5287.17X - 9343.80	0.9999
PHBA	Y = 30159.2X + 181.521	0.9999
HBD	Y = 34078.1X - 3806.87	0.9999
PE	Y = 2253.15X - 11206.5	0.9999
PB	Y = 1006.62X - 3790.12	0.9999
PC	Y = 666.944X + 55.8872	0.9999
PA	Y = 1013.37X + 5092.17	0.9999

Table S4 Monosaccharide component regression curve parameters

Detection Indicators	Peak Time	Slope	Fit Degree
Ara	10.2087	1.7452	0.9961
Gal	13.0670	1.5981	0.9973
Glc	15.0753	1.6103	0.9985
Gal-UA	34.1420	0.4823	0.9938

Table S5 Content of main active ingredients (mg·g^{-1}) in different groups of *Gastrodia elata* (n=3)

Group	GAS	HBA	PHBA	HBD	PE	PB	PC	PA	Total P	Output (kg·m^{-2})
A	0.923	1.249	0.016	0.182	1.396	5.061	1.453	12.168	20.078	1928.12
A	0.945	1.242	0.017	0.188	1.398	5.068	1.469	12.174	20.109	1974.52
A	0.952	1.236	0.018	0.193	1.285	5.079	1.476	12.186	20.026	1962.86
B	0.743	0.937	0.039	0.171	0.978	4.942	1.246	9.306	16.472	3375.48
B	0.738	0.940	0.036	0.178	0.970	4.935	1.252	9.313	16.470	3679.5
B	0.731	0.942	0.029	0.184	0.969	4.928	1.267	9.321	16.485	4194.4
C	0.554	0.994	0.028	0.229	1.224	4.659	1.243	9.359	16.485	2623.52
C	0.568	0.992	0.029	0.223	1.218	4.669	1.259	9.369	16.515	3402.28
C	0.569	0.989	0.028	0.217	1.209	4.671	1.267	9.378	16.525	2807.94
D	0.443	0.681	0.065	0.229	1.098	4.084	1.306	8.549	15.037	2128.44
D	0.449	0.670	0.061	0.225	1.088	4.078	1.291	8.541	14.998	1452.56
D	0.452	0.673	0.059	0.216	1.064	4.068	1.287	8.492	14.911	1938.76
E	0.441	0.768	0.043	0.199	0.910	4.311	1.104	7.281	13.606	3021.88
E	0.444	0.770	0.042	0.191	0.918	4.302	1.099	7.298	13.617	3206.14
E	0.449	0.773	0.039	0.186	0.926	4.298	1.087	7.308	13.619	2268.36
